# Myoepithelioma of the larynx: a case report

**DOI:** 10.4076/1757-1626-2-8085

**Published:** 2009-08-26

**Authors:** Angelos Chatziavramidis, Alexandra Grekou, Ioannis Thomaidis, Thomas Sidiras

**Affiliations:** 1 ENT, Head & Neck Surgery Clinic, “Theagenion” Oncologic HospitalAlexandros Symeonidis Str. 2, 54007 ThessalonikiGreece; 2 Pathology Department, “Theagenion” Oncologic HospitalAlexandros Symeonidis Str. 2, 54007 ThessalonikiGreece

## Abstract

Myoepithelioma of the larynx is a very rare tumor with nonspecific local symptoms. We present the second known case, focusing on the peculiarities of the differential diagnosis for this type of tumor that are crucial for the right histologic diagnosis and furthermore for the therapeutic outcome.

We report a 37-year-old male presenting with hoarseness and dyspnea. The indirect laryngoscopy revealed a gross glottic tumor from the right vocal cord who occupied the greater part of the glottis. No apparent cartilage invasion was shown in the CT. He came to us with a previous direct laryngoscopy derived biopsy describing a chondroma. A modified vertical partial laryngectomy, under temporary tracheostomy, with muscle reconstruction for the deficit of the right vocal cord was applied for the removal of the tumor. The final histopathologic diagnosis was myoepithelioma (spindle cell type) of the larynx. A long term follow-up in our case showed no recurrence and a good functional result.

The larynx is a very rare localization for this type of tumour. The benign character of the disease in conjunction with its slow progression could delay its detection and diagnosis, leading to a more destructive surgery. A detailed pathology examination is prerequisite for avoidance of misleading diagnosis.

## Introduction

Myoepithelioma is a rare salivary gland tumor arising from proliferation of myoepithelial cells.

Sheldon initially published his work on a myoepithelioma in 1943 [1], and first in 1991 and later in 2005 myoepithelioma was recognized from the WHO as a distinct entity [2,3].

These tumours represent 1%-1.5% of all salivary gland tumours, and are distributed 48% in parotis, 42% in the small salivary glands and the remaining in glandula submandibularis and seromucous glands of the nose and Larynx [4]. Other localizations reported are the skin, chest, lung and pancreas [4,5].

The tumour shows no gender preference and is more frequent in the 3rd decade of life [3].

According to our medline research for keywords “larynx, myoepithelioma”, only one benign case and one concerning a malignant tumour with liver metastases have been published [6,7]. Therefore we present the second benign larynx localization of myoepithelioma.

## Case presentation

A 37-year-old Greek man presented in our out-patient office, complaining of slowly aggravating hoarseness for the last two years and activity-dyspnea coexisting for the last two months. There was no pain, dysphagia or general symptom.

One and a half years ago he underwent microlaryngoscopy under general anaesthesia at a different institution due to the presence of an endolaryngeal expanding mass. Biopsy was taken from the tumour and the histological findings concerned a larynx chondroma.

Fiberoptic endoscopy (70°) of the larynx revealed a well defined intraluminal expanded tumour arising from the right hemilarynx, which was covered with normal mucosa. Mobility of the right side was reduced. The remaining structures of the larynx showed unremarkable findings. Neck palpation was negative for lymph nodes.

**Figure 1.**
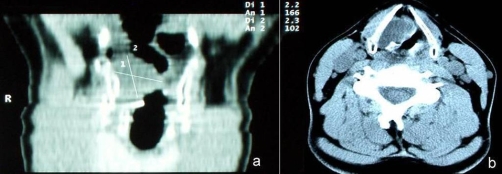
Preoperatively **(a)** coronal and **(b)** axial CT sections of the larynx to the level of the true vocal cords showed an exophytic tumour, which occupied the largest part of the glottis extending from the right arytenoid region to the anterior third of the left vocal cord.

**Figure 2.**
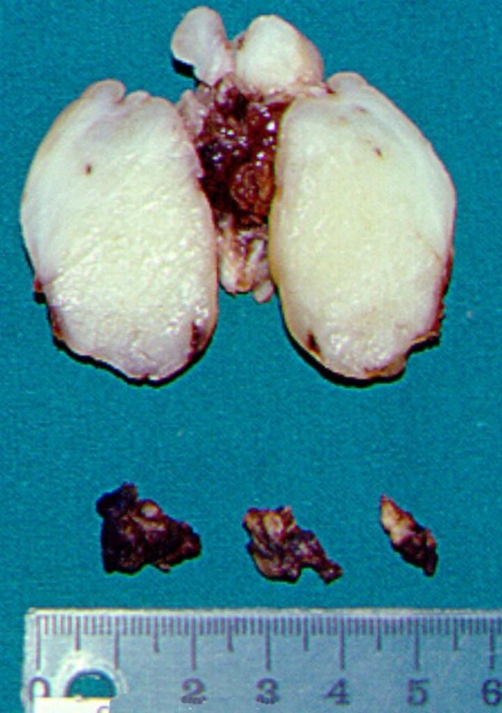
The 5.0 cm × 2.8 cm × 2.0 cm surgical specimen resulted after a laryngofissure (modified Leroux Robert technique) approach. It consisted of the en-block resected tumour with the right true and false vocal fold, the anterior commissure, the anterior third of the left vocal fold and the right subglottic area, and also part of the arytenoid cartilage. A 1,3 cm wide vertical strip was excised from the anterior right thyroid cartilage. Central biopsies were taken from right arytenoid region, right subglottic space and anterior edge of left vocal fold (bottom of the figure).

CT scan of the neck showed a soft mass glottic tumour, expanding from the right arytenoid to the anterior third of the left true vocal cord occupying the greater part of glottis (Figure 1). A laryngofissure (modified Leroux Robert technique) was performed under temporary tracheostomy. The tumour was an-block resected with preservation of the largest part of the right arytenoid and thyroid cartilage. The extent of the specimen may be observed in (Figure 2).

The laryngeal wall deficit was reconstructed with a loco regional flap from ipsilateral sternohyoideus muscle in conjunction with thyroid cartilage outer perichondrium.

The patient recovered uneventfully and tracheostomy was closed 3 weeks post-op.

**Figure 3.**
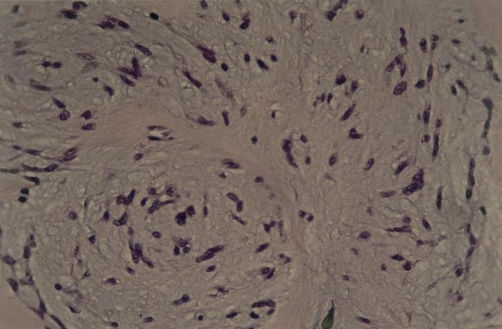
Histological section of the tumour showed a laryngeal myoepithelioma with spindle-shaped cells loosely arranged into knot formations, which were well defined with fine strips of hyaline supporting tissue (HE × 200).

**Figure 4.**
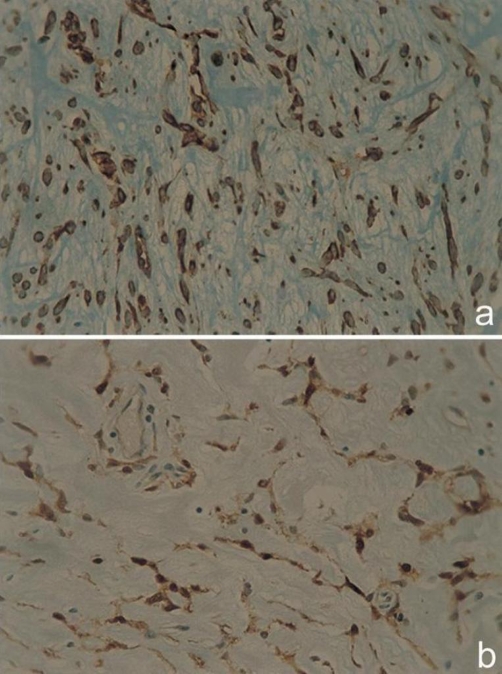
Immunohistochemistry showed **(a)** positive cytoplasm immune reaction of the neoplastic cells for actin- muscle specific, **(b)** positive cytoplasm and nuclear immune colouring of the neoplastic cells for S-100 protein. Cells with dendritic projections were also recognized. (PAP/DAB × 400).

Histology revealed spindle shaped cells loosely arranged in knot formations, separated by fine strips of hyaline extracellular stroma tissue, and also cells without atypical nuclear mitotic activity laid in loose microcystic and myxoidic degenerated collagenstroma. Further immunohistochemical studies were positive for S-100 protein, vimentin and actin muscle specific (Figures 3 and 4). Diagnosis: benign myoepithelioma (spindle cell type) of the larynx.

**Figure 5.**
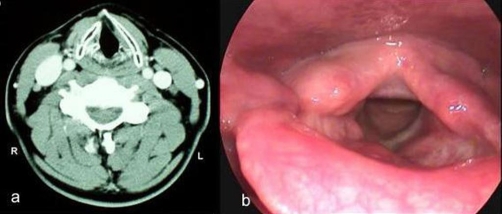
**(a)** Axial CT section of the neck to the level of true vocal cords and **(b)** rigid fiberoptic (70 °) endoscopy more than 8 years post operatively showed well epithelized surface of the surgical defect at the right side of the larynx. There is no evidence for a recurrence.

Postoperative long term follow up (> 8 years) either with fiberoptic laryngoscopy or with CT was uneventful (Figure 5), which ensures the accuracy of histology and the total resection of the tumour.

By self assessment the patient reported a mild deterioration of his voice not disturbing his social or professional life, and the Voice Handicap Index (VHI) resulted a total value 16 (F = 2, P = 11, E = 3).

## Discussion

Myoepithelioma of the larynx is a very rare tumour, originating from the myoepithelial cells of larynx mucosa glands. These cells containing myofilaments in their cytoplasm show contractility, they support the parenchyma, and contribute to the production of laminin, collagen type-IV and fibronectin to maintain the “basal lamina” [6]. The increasing myoepithelial cells are arranged in bound -, nests -, or mandel like structures. The presence of spindle cells, plasmacytoid, epithelioid, “clear” cells or combinations determines the histological classification of the tumour respectively [3,5,8,9]. Myoepitheliomas are slowly growing, well encapsulated and defined masses [4]. They are generally differentiated from pleomorphic adenomas by means of absence or minimum participation (<5%-10%) of ductal and acinic components [3,8,10-12].

A large majority of myoepitheliomas are of benign character. Malignant tumours (10%) show a destructive-infiltrative growth pattern with necroses, rough chromatin, marked cellular pleomorphism, and high mitotic activity [5,7,11].

Some cases could present unusual difficulties in macro and microscopic diagnosis.

Electronic microscopy could be of importance for the identification of cytoplasmic myofilaments and pinocytic vesicles [6,7]. Further immunohistochemical diagnostic assays include positive S-100 protein, actin smooth muscle specific, cytoceratin 14, cytoceratin, vimentin and fibrous glycoproteins [8].

The most frequent types of myoepithelioma are: the plasmocytoid-, and the spindle-cell form. Differential diagnosis for the plasmocytoid form comprises metastatic carcinoma with plasmocytoid morphology, oncocytic adenoma and melanoma [5]. The spindle-cell form has to be differentiated from mesenchymal tumours (smooth musculature tumours, schwannomas, and fibroblastic tumours) and amelanotic melanomas [5].

In our case, a benign spindle-form myoepithelioma, the pathologist though sceptical was led to exclude the following differential diagnostic entities:

a) Pleomorphic adenoma: after thorough macro - microscopic as well as immuno-histochemical investigation no epithelial elements were proven, b) tumour of the cartilaginous larynxskeleton (chondroma, chondrosarkoma): no typical cartilaginous regions, as well as no atypical nuclei, necrosis, mitotic activity or infiltrative expansions were found, c) malignant myoepithelioma: in the periphery of the tumour nodular elongations were observed, which are compared to the histology of pleomorphic adenoma of the salivary glands. The absence of definite infiltrative growth, as well as the benign features of the neoplastic cells supported a benign biological behaviour.

Treatment of choice for the benign lesions is excision within a zone of healthy surrounding tissue. The malignant tumours necessitate broadly tumour-free margins, neck-dissection and radiotherapy [5].

## Conclusion

The larynx constitutes a very rare localization for this benign tumour, with this case being the second published myoepithelioma of the larynx. Peculiar differential diagnostic problems concerning the histology should be approached with a spectrum of available methods targeting to the final result. The character and the expansion of the lesion will determine the therapeutic approach and the final outcome respectively.
